# Counterterrorism policies and practices: health and values at stake

**DOI:** 10.2471/BLT.14.144816

**Published:** 2015-08-31

**Authors:** Lisa Eckenwiler, Matthew Hunt, Ayesha Ahmad, Philippe Calain, Angus Dawson, Robert Goodin, Daniel Messelken, Leonard Rubenstein, Verina Wild

**Affiliations:** a Philosophy and Health Administration and Policy, George Mason University, 4400 University Avenue, Fairfax, Virginia, 22030, United States of America (USA).; b School of Physical and Occupational Therapy and Researcher, McGill University, Montreal, Canada.; c University College London, London, England.; d Research Unit on Humanitarian Stakes and Practices, Médecins Sans Frontières, Geneva, Switzerland.; e Centre for Values, Ethics, and the Law in Medicine, University of Sydney, Australia.; f Australian National University, Canberra, Australia.; g Center for Ethics, University of Zurich, Switzerland.; h Program on Human Rights and Health in Conflict, Johns Hopkins Bloomberg School of Public Health, Baltimore, USA.; i Department of Philosophy, Ludwig-Maximilians-University, Munich, Germany.

The United States Central Intelligence Agency (CIA) used a fake vaccination programme to obtain DNA (deoxyribonucleic acid) samples in the search for Osama Bin Laden, which caused distrust and hampered polio eradication and other public health efforts in Pakistan.^1^^,^^2^ The Obama administration’s vow that the CIA will never again exploit a vaccination programme in its counterterrorism efforts, therefore came as welcome news to global health and humanitarian communities.^3^

Distrust and suspicion that public health programmes are being used to advance foreign interests have contributed to the increase in murders and violent attacks on vaccination workers.^2^ There have been setbacks to polio eradication efforts and other public health objectives.^2^ Counterterrorism policies and practices can have unintended health impacts, especially where health programmes are co-opted or undermined, in countries where health systems are strained and population-health indicators are poor.

The reach of counterterrorism laws is long and they have adversely affected humanitarian health activities in many countries where identified terrorist groups are active and health needs are increased.^4^ Humanitarian actions can be categorized as providing material support to terrorists. Material support has been interpreted to include the provision of medical care (but not medicines), which can render the very activities that are associated with the core ethical commitments of the medical and nursing professions illegal.

Even where specific prohibitions are not in place, such policies have a range of more diffuse effects which can undermine population health. Humanitarian organizations have become more hesitant to rely on local contractors who once provided essential resources like transportation and equipment for fear of making them vulnerable to criminal prosecution or violence.^4^ Risks of violence have, indeed, increased for health providers where local populations and armed factions perceive them as neither neutral nor impartial, and ultimately untrustworthy.^1^

This situation contributes to rising security concerns for health providers and facilities.^5^ The greatest risks, alongside adverse impacts on population health, are incurred by local health workers who may be seen as betraying their own communities, or perceived by other groups to be enemies for having treated members of those communities. Local health workers are typically unable to leave their communities in the face of danger and have access to fewer protections, compared to expatriate humanitarian workers.^6^

Intelligence officials may attempt to use health organizations and workers to gather intelligence. The United States military has also used health care in the context of counterinsurgency operations.^7^ These counterterrorism policies and practices can threaten people’s health by creating the conditions for distrust and by deterring people from seeking care. For humanitarian health workers, the principles of impartiality and independence, which lie at the centre of humanitarian work, are undermined. This can lead to moral distress for health workers concerning accountability to intended beneficiaries of services and to funders, responsibility to patients and the law, complicity with perceived wrongs and compromise of professional and personal ethical commitments.^6^^,^^8^

Several ethical values and principles are at stake, including: trust, solidarity, proportionality and accountability. Trust is an essential aspect of all human social interaction, but is especially important in global health work, where health workers employed in a particular public health programme have not previously worked with the local population. Solidarity, although traditionally interpreted as a principle and practice embraced within the confines of community, is now global in scope. Solidarity involves cultivating bonds with others, trying to imagine their plight and standing with them in fighting injustice. In advancing their counterterrorism agenda, strategists and policy-makers should not threaten solidarity in global health action. Indeed, we have witnessed solidarity around the moral imperative to detach counterterrorism measures from health programmes and interventions.^2^^,^^3^

The principle of proportionality states that there should be a balance between the risks of harm and the potential benefits of a given intervention. In this context there is no evidence that population-health impacts are considered by security advisers, an oversight we find ethically unjustifiable given the potential for harm resulting from decisions on the methods used to combat terrorism. This omission also violates obligations to respect and protect health care, established under international humanitarian law^9^ and human rights law.^10^

Those focused on fighting terrorism have the responsibility of weighing the potential health consequences for people living in areas targeted by counterterrorism efforts. To the extent that counterterrorism operations, laws, and policies damage population health – especially where these effects are foreseeable and preventable – such responsibilities are clearly established in ethics and international law.

New mechanisms to ensure that counterterrorism activities do not contravene international law or ethical values and principles will require careful design. Apart from the ethical and legal grounds, there are good practical reasons to design more effective counterterrorism measures. Preventable harms to population health contribute to mistrust and instability and undermine the stated objectives of the intelligence services.
